# Interferon-Induced Protein 44 Correlated With Immune Infiltration Serves as a Potential Prognostic Indicator in Head and Neck Squamous Cell Carcinoma

**DOI:** 10.3389/fonc.2020.557157

**Published:** 2020-10-06

**Authors:** Hua Pan, Xiaoqing Wang, Weiqiang Huang, Yongmei Dai, Mi Yang, Huazhen Liang, Xixi Wu, Longshan Zhang, Wenqi Huang, Lu Yuan, Yuting Wu, Yin Wang, Liwei Liao, Jihong Huang, Jian Guan

**Affiliations:** ^1^Department of Radiation Oncology, Nanfang Hospital, Southern Medical University, Guangzhou, China; ^2^Department of Oncology, Fujian Provincial Hospital, Fuzhou, China; ^3^Department of Oncology, Maoming People’s Hospital, Maoming, China

**Keywords:** IFI44, head and neck squamous cell carcinoma, poor prognosis, immune infiltration, locally advanced

## Abstract

Interferon-induced protein 44 (IFI44) containing a guanosine-5′-triphosphate (GTP) binding domain was reported to play a significant role in the immune response to autoimmune disease. However, its roles involved in cancers remain unclear. Here, we detected the expression of IFI44 in The Cancer Genome Atlas (TCGA) Pan-cancer and generally explored the effect of IFI44 on immune infiltration in the tumor microenvironment (TME). The results displayed that IFI44 was mainly located in the cytoplasm and overexpressed in head and neck squamous cell carcinoma (HNSC) samples compared with normal tissues. Survival analysis exhibited that IFI44 was remarkably associated with the clinical outcomes, particularly in lymph node-positive and locally advanced HNSC patients. Biological analysis showed that IFI44 was correlated with such immune biological processes as antigen-presenting and nuclear factor (NF)-kappa B signaling pathways. Immune signature analysis demonstrated that the expression of IFI44 was positively correlated with the infiltration of CD4^+^ cells and macrophages as well as neutrophils in HNSC. Taken together, these data suggested that IFI44 was abnormally expressed in cancer tissues and indicated the potential impact of IFI44 on the tumor immune infiltration in HNSC.

## Introduction

As the seventh most common cancer, head and neck squamous cell carcinoma (HNSC) covers those subcategories that originate from the nasal cavity, sinuses, nasopharynx, oropharynx, laryngopharynx, cervical esophagus, salivary glands, mouth, throat, and ear (1). In the year 2018, the number of HNSC new cases is near 890,000, and about 450,000 people died from HNSC annually (2). Unfortunately, more than half of HNSC patients are initially diagnosed at advanced stages (2, 3). After surgery or chemoradiotherapy, there are still over 50% of patients having recurrence or metastasis with poor clinical prognosis (4). Therefore, it is urgent to develop more effective therapeutic strategies for HNSC patients. The occurrence and mechanisms of tumors are complex and multi-caused, among which immune-related mechanisms play a crucial role in the development of HNSC (3, 5). Currently, immune checkpoint inhibitor-targeted drugs, for example, pembrolizumab, have been applied to the first-line treatment of recurrent/metastatic HNSC patients (6, 7). However, a large proportion of HNSC patients have failed to respond to immune checkpoint block (ICB) therapies (5, 8). Thus, it is urgent to identify the appropriate subtypes of HNSC suitable for immune checkpoint therapy to amplify clinical benefit and enhance the antitumor effect.

Interferon-induced protein 44 (IFI44) is the product of the protein-coding gene located at 1p31, originally found to affect the formation of microtubular structures in the study of hepatitis C virus (HCV) infection (9, 10). This gene mRNA transcript consists of four coding sequences (CDSs) acquired from an online tool, GenBank^1^. Comparative genomics analysis shows that IFI44 contains a guanosine-5′-triphosphate (GTP) binding site and has no homology to GTPases or G proteins (11). IFI44 is required to negatively regulate the host antiviral responses and autoimmunity due to its crucial roles in innate immune responses (12). In a previous study, IFI44 has been found to suppress the proliferation of two human melanoma cell lines (11). Recently, Wang et al. (13) has pointed out the mechanisms mediated by IFI44 to reverse the resistance to gefitinib in non-small-cell lung cancer-targeted therapy. However, its expression and related infiltration of immune cells in other cancers are unidentified.

In our study, we analyzed the differential expression of IFI44 in tumors and paired adjacent or normal tissues through The Cancer Genome Atlas (TCGA) and Gene Expression Omnibus (GEO) databases. Based on different IFI44 expressions at the mRNA level, we discovered its potential impacts on HNSC. Survival analysis exhibited that IFI44 had a good predictive value in metastasis and recurrence for locally advanced HNSC. The Kyoto Encyclopedia of Genes and Genomes (KEGG) and Gene Ontology (GO) results showed that IFI44 was correlated with tumor formation and progression. In addition, immune infiltration associated with IFI44 mRNA expression was explored through Tumor IMmune Estimation Resource (TIMER) and Estimation of STromal and Immune cells in MAlignant Tumors using Expression data (ESTIMATE) databases. With the significant prognostic roles of IFI44 in HNSC, multiple analyses were performed, including competing endogenous RNA (ceRNA) network, methylation, copy number count and tumor mutation burden. Our initial exploration demonstrated that IFI44 perhaps closely correlated with immune infiltration in the tumor microenvironment (TME), which contributed a lot to the tumorigenesis and development of HNSC.

## Materials and Methods

### Human Specimen Collection

A total of 65 human tissue samples were collected from Nanfang Hospital (Guangzhou, China) from January 2017 to January 2018, among of which 21 paired tumor and normal specimens were enrolled. The anatomical sites of collected samples included head and neck (mouth, tongue, nasopharynx, and oropharynx), colorectal, uterus, cervix, liver, breast, and stomach. All of these involved patients have never undergone chemoradiotherapy, radiotherapy, chemotherapy, targeted therapy, and immune-associated therapy before resection. All collected specimens have been approved by the Nan Fang Hospital Institutional Review Board.

### Gene Expression, Genetic and Epigenetic Level Analysis in Different Databases

Having analyzed the mRNA expression of IFI44 in multiple cancer patients through TCGA Program^2^ and GEO^3^ databases, we found its abnormal expression in the tumor section in comparison with normal tissues or adjacent normal tissues. The methylation beta value of IFI44 and all (microRNAs) and long-chain non-coding RNA (LncRNA) expression data of TCGA HNSC samples were retrieved from UCSC database^4^. The mutation count and copy number count of TCGA HNSC cases were downloaded from cBioportal database^5^.

### Gene Expression Profiling Interactive Analysis Database

The prognostic value of IFI44 in multiple cancer patients was evaluated *via* Gene Expression Profiling Interactive Analysis (GEPIA^6^) database. GEPIA is an online RNA-sequencing (RNA-seq) data analysis platform where all results are calculated using TCGA data. The survival analysis of IFI44 mRNA expression in cancers included overall survival (OS), disease-specific survival (DSS), progression-free interval (PFI), and disease-free survival (DFS). The survival curves of different tumors were presented in the figure with the remarks of log-rank test *P*-value and the number of patients in each group. Further, the top 100 of IFI44 co-expression genes in HNSC tumor and normal samples were, respectively obtained from the GEPIA database.

### Immunohistochemistry

Both tumor samples and matched normal tissues were resected from patients within 24 h. After being fixed in 10% paraformaldehyde, washed, dehydrated, and embedded in sequence, all tissue samples were sliced into 3-μm pieces. Ethylenediaminetetraacetic acid (EDTA) solution was used for tissue antigen retrieval in a pressure cooker for 10 min after boiling. Endogenous peroxidase activity was blocked with 3% H_2_O_2_ for 15 min at room temperature. Then, sections were incubated with primary monoclonal human/mouse IFI44 antibody at 4°C overnight. The antibody was purchased from Proteintech Group, Inc. The operation process was strictly based on the instructions. The expression level and location of IFI44 were observed by two observers independently.

### Gene Ontology and Kyoto Encyclopedia of Genes and Genomes Analysis

Gene expression Profiling interactive analysis co-expression analysis produced IFI44 co-expression gene set, respectively in HNSC tumor samples and normal samples, among which 60 genes uniquely co-expressed in the tumors were selected to analyze the underlying mechanism of IFI44 in HNSC. The selected gene set was uploaded to the Database for Annotation, Visualization, and Integrated Discovery (DAVID Version 6.8), a functional annotation tool^7^ for analyzing GO and KEGG pathway. Cytoscape software 3.7.1^8^ was further utilized to verify the KEGG pathway analysis results. All exhibiting results were filtered with the standard of *P*-value less than 0.05.

### Gene Set Enrichment Analysis

Gene Set Enrichment Analysis (GSEA Version 4.0.3) was a Java desk application software (downloaded from http://software.broadinstitute.org/gsea/index.jsp). Firstly, we predefined c7 immune signature as the background gene set, which was downloaded from the MSigDB database of the GSEA website. Further, we classified the HNSC tumor samples from TCGA database into two groups according to IFI44 mRNA expression (cutoff = median IFI44 mRNA expression) and then tested whether the preset genes were at the top or bottom of the sorted table. The result was calculated after 1,000 cycles. A series of gene sets were selected based on the standard *P*-value < 0.05 and false discovery rate (FDR) *P*-value < 0.25.

### Immune Cell Infiltration Analysis

TIMER2.0 is a web portal evaluating the interaction between the tumor and the immune system, which integrates multiple heterogeneous types of data^9^. The TIMER2.0 database not only stores gene-associated data including gene symbol, name, location, and the relationship between current gene and antitumor immunity but also provides infiltration estimation data related to all TCGA tumor samples for users across TIMER, CIBERSORT, quanTIseq, xCell, MCPCounter, and Estimating the Proportion of Immune and Cancer cells (EPIC) database (14–19). The ESTIMATE database was utilized to figure out the immune-associated score of input samples (20). We used the TIMER database to analyze the relationship between the levels of immune infiltration and the expression of the enriched immune gene set in HNSC. We analyzed immune infiltration data including different types of immune cells associated with IFI44 mRNA expression. The composed proportions of immune cells in the two groups were computed using average score, and the significance was based on the *t*-test.

### Statistical Analysis

Graph prism 7.0 software was employed to compare the differential mRNA expression between tumor and paired normal samples, where *P*-value was counted with the *t*-test. All survival analysis associated with different IFI44 mRNA expression was also completed by using Graph prism 7.0. The calculation of *P*-value was acquired *via* log-rank test in Table 1. Hazard ratio (HR) and 95% confidence interval (CI) were evaluated by Mantel–Haenszel. The best expression cutoff of IFI44 to divide HNSC patients into two groups was calculated through X-Tile software. The survival curves from GEPIA were exhibited with HR and *P*-value. Different immune infiltration landscapes were established by Excel 2016. Differential miRNAs, LncRNAs, and mRNAs were all selected using Excel 2016.

**TABLE 1:** The correlation of IFI44 mRNA-seq data with the survival analysis in HNSC patients.

clinical characteristics	Overall survival			Progression-free interval			Disease-specific survival		
			
	*N*	Hazard ratio	*P*-value	*N*	Hazard ratio	*P*-value	*N*	Hazard ratio	*P*-value
**Stage**									
I	18	1.022(0.2055∼5.086)	0.9784	18	2.156(0.4321∼10.76)	0.3488	18	1.467(0.2534∼8.496)	0.6687
II	95	0.9961(0.5333∼1.861)	0.9902	95	1.395(0.7022∼2.771)	0.342	88	0.8903(0.3754∼2.111)	0.792
III	99	0.6783(0.371∼1.24)	0.2074	99	0.5168(0.2614∼1.021)	0.0576	92	0.4796(0.2134∼1.078)	0.0753
IV	266	1.906(1.315∼2.764)	**0.0007**	266	2.084(1.419∼3.062)	**0.0002**	255	2.392(1.491∼3.839)	**0.0003**
I+II	113	0.9631(0.5387∼1.722)	0.899	113	1.343(0.7157∼2.519)	0.3586	106	0.8682(0.4012∼1.879)	0.7198
III+IV	365	1.492(1.09∼2.044)	**0.0126**	365	1.482(1.065∼2.063)	**0.0198**	347	1.661(1.108∼2.489)	**0.0141**
**Stage T**									
1	31	0.9425(0.2352∼3.777)	0.9334	31	1.345(0.3053∼5.923)	0.6955	31	1.36(0.235∼7.874)	0.7312
2	141	0.9442(0.5523∼1.614)	0.8337	132	0.9884(0.5571∼1.754)	0.9683	131	0.6272(0.2904∼1.355)	0.2351
3	128	1.012(0.6109∼1.675)	0.9645	128	0.8991(0.5243∼1.542)	0.6989	121	0.9246(0.5077∼1.684)	0.7978
4	178	1.878(1.204∼2.931)	**0.0055**	178	1.782(1.121∼2.832)	**0.0145**	170	1.956(1.099∼3.481)	**0.0226**
**Stage N**									
0	240	0.8419(0.57∼1.244)	0.3874	240	1.027(0.6725∼1.567)	0.9033	225	0.7921(0.4641∼1.352)	0.3926
1	78	1.071(0.548∼2.095)	0.8402	78	0.9992(0.4598∼2.171)	0.9983	72	0.8882(0.3476∼2.27)	0.8043
2	151	2.568(1.56∼4.228)	**0.0002**	151	2.103(1.275∼3.467)	**0.0036**	148	2.844(1.573∼5.141)	**0.0005**
3	9	2.506(0.4201∼14.96)	0.3133	9	2.465(0.4114∼14.77)	0.3233	8	2.549(0.3473∼18.7)	0.3576
1+2+3	238	1.932(1.309∼2.851)	**0.0009**	238	1.736(1.156∼2.608)	**0.0078**	228	2.22(1.368∼3.602)	**0.0012**
**Stage M**									
0	478	1.31(0.9956∼1.725)	0.0538	478	1.323(0.9872∼1.772)	0.061	453	1.28(0.8959∼1.829)	0.1751
**Histologic grade**									
1	62	1.551(0.6672∼3.606)	0.3078	62	1.686(0.6868∼4.138)	0.2543	59	1.008(0.3177∼3.201)	0.9886
2	303	1.063(0.7553∼1.496)	0.7264	303	1.238(0.8678∼1.767)	0.2387	291	1.157(0.7459∼1.794)	0.5155
3	125	1.638(0.9565∼2.806)	0.0722	125	1.239(0.7015∼2.188)	0.4605	119	1.301(0.6742∼2.512)	0.4325

*The bold P value represents statistical significance.*

## Results

### The Expression Profile of IFI44 in Cancers

**FIGURE 1:**
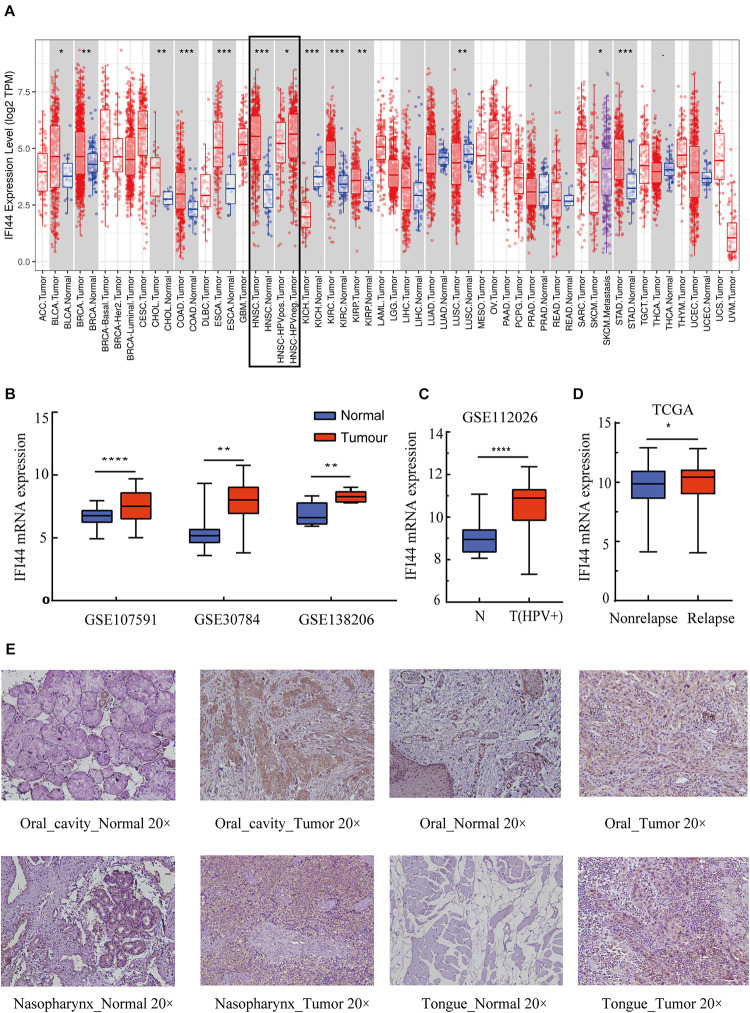
The different IFI44 expression in multiple cancers. **(A)** The different IFI44 expression in TCGA Pan-cancer samples including tumor and normal tissues analyzed by TIMER database. **(B)** The overexpression of IFI44 in head and neck squamous cell carcinoma (HNSC) tumor section was confirmed in three GEO datasets (GSE107591, 45 normal vs. 167 cancer, *P* < 0.0001; GSE30784, 23 normal vs. 24 cancer, *P* = 0.0076; GSE138206, 12 normal vs. 6 cancer, *P* = 0.0023). **(C)** Human papillomavirus (HPV)-positive oral cavity carcinoma (OCC) expressed higher IFI44 than normal samples (GSE112026, 25 normal vs. 47 cancer, *P* < 0.0001). **(D)** IFI44 was highly expressed in HNSC relapsed samples than non-relapsed patients (non-relapsed, *n* = 351; relapsed, *n* = 133, *P* = 0.0315). **(E)** Expression of IFI44 proteins in different tumor tissues and paired normal tissues in HNSC by immunohistochemistry assay. (P-value significant codes: *P < 0.05; **P < 0.01; ***P < 0.001; ****P < 0.0001).

The cancer genome atlas pan-cancer RNA-seq datasets were analyzed to compare IFI44 expressions across different types of tumors and adjacent normal tissues. The results exhibited that IFI44 was significantly differently expressed in such cancers as HNSC, bladder urothelial carcinoma (BLCA), breast invasive carcinoma (BRCA), cholangiocarcinoma (CHOL), colon adenocarcinoma (COAD), esophageal carcinoma (ESCA), kidney chromophobe (KICH), kidney renal papillary cell carcinoma (KIRP), liver hepatocellular carcinoma (LIHC), lung adenocarcinoma (LUAD), lung squamous cell carcinoma (LUSC), and stomach adenocarcinoma (STAD) (Figure 1A). Further, HNSC tumor sections demonstrated a higher expression of IFI44 than the paired normal tissues (Figure 1A; *P* < 0.001). Besides, the overexpression of IFI44 in the HNSC section was verified in three public microarray datasets (Figure 1B; GSE107591, 45 normal vs. 167 cancer, *P* < 0.0001; GSE30784, 23 normal vs. 24 cancer, *P* = 0.0076; GSE138206, 12 normal vs. 6 cancer, *P* = 0.0023). Consistently, analysis of human papillomavirus (HPV) positive oropharyngeal squamous cell carcinoma showed the same distribution of IFI44 expression (Figure 1C; GSE112026, 25 normal vs. 47 cancer, *P* < 0.0001). Remarkably, the lower expression of IFI44 was observed in TCGA HPV-positive tumors than negative samples (Figure 1A, *P* = 0.0138). The relapsed cases expressed higher IFI44 than non-relapsed patients (Figure 1D, *P* = 0.0315).

To clarify the different expressions of IFI44 at the protein level, immunohistochemistry experiments were conducted in human tumor samples and normal tissues. All results exhibited that IFI44 protein was mainly located in the cytoplasm and widely expressed in several human cancer sections and normal tissues (Figure 1E and Supplementary Figure 1). In hepatocellular carcinoma, IFI44 presented a slightly higher expression in tumor samples than paired normal tissues. In contrast, the protein of IFI44 showed lower expression in gastric cancer. In the case of cervical squamous cell carcinoma, the expression of IFI44 was observed to be higher in tumor parenchyma compared with the stroma region. In HNSC, IFI44 expression was moderately higher in tumor samples than that in normal samples in general. In particular, increased expression of IFI44 was found in laryngeal, nasopharyngeal, tongue, and oral carcinoma. Together, the immunohistochemistry results demonstrated the same upregulation of IFI44 in head and neck and cervical squamous cell carcinoma.

### Prognostic Potential of IFI44 in Cancers

Based on the present research, the analysis of survival time was conducted to clarify the relationship between IFI44 expression and the prognosis in multiple malignancies *via* the GEPIA database. The results from GEPIA (Supplementary Figure 2) exhibited that IFI44-high expression group was significantly associated with shorter OS and DFS compared with IFI44-low expression group in HNSC (*P* = 0.022 and HR = 1.5 for DFS), brain lower grade glioma (LGG) (*P* = 2.1e−07 and HR = 2.6 for OS; *P* = 5.1e−06 and HR = 2 for DFS), prostate adenocarcinoma (PRAD) (*P* = 0.0035 and HR = 1.9 for DFS), uveal melanoma (UVM) (*P* = 0.026 and HR = 2.8 for OS; *P* = 0.031 and HR = 2.8 for DFS), thymoma (THYM) (*P* = 0.017 and HR = 8.4 for OS). Instead, IFI44-high expression was associated with longer DFS in Uterine Carcinosarcoma (UCS) (*P* = 0.023 and HR = 0.44 for DFS) and OS in skin cutaneous melanoma (SKCM) (*P* = 0.0027 and HR = 0.67 for OS) patients.

**FIGURE 2:**
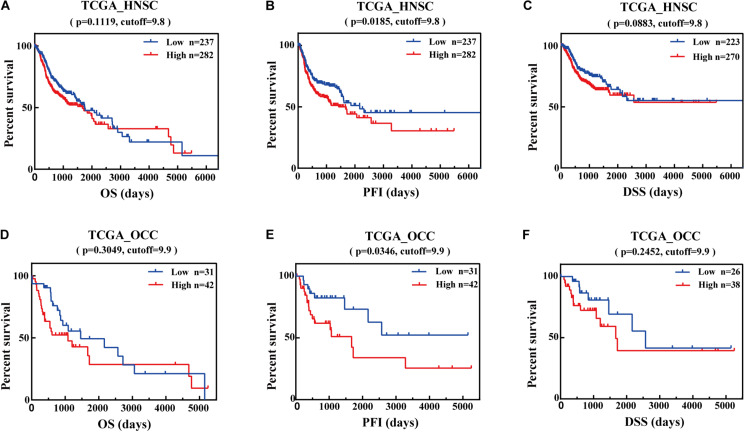
The prognostic potential value of IFI44 in head and neck squamous cell carcinoma (HNSC). **(A)** Overall survival (OS), **(B)** progression-free interval (PFI), and **(C)** disease-specific survival (DSS) of two different expression levels of IFI44 in HNSC. **(D)** OS, **(E)** PFI, and **(F)** DSS of two different expression levels of IFI44 in oral cavity carcinoma (OCC).

Further, we utilized the data from TCGA database to analyze the OS, DSS, and PFI associated with different IFI44 expression levels in HNSC. The results were consistent with the analysis from GEPIA that IFI44-high expression group had the same poor prognosis in HNSC (Figures 2A–C; OS, *P* = 0.1119, cutoff = 9.8; PFI, *P* = 0.0185, cutoff = 9.8; DSS, *P* = 0.0883, cutoff = 9.8). Relatively, the prognosis of oral cavity carcinoma (OCC) was closely related to IFI44 mRNA expression level. Figures 2D–F displayed that IFI44-high expression group has poor OS, DSS, and PFI in OCC (*P* = 0.2452, cutoff = 9.9 for OS; *P* = 0.0346, cutoff = 9.9 for PFI, *P* = 0.2452, cutoff = 9.9 for DSS). The data implied that the potential prognostic value of IFI44 deserves to be continusouly investigated in HNSC patients.

Therefore, we evaluated the relationship between IFI44 mRNA expression and different clinical stages of HNSC patients from TCGA database. Patients with unclear clinical properties or initially diagnosed with metastasis were excluded. As shown in Table 1, overexpression of IFI44 was significantly associated with worse OS, PFI, and DSS, especially in Stage IV (*n* = 266, HR = 1.906, *P* = 0.0007 for OS; *n* = 266, HR = 2.084, *P* = 0.0002 for PFI; *n* = 255, HR = 2.392, *P* = 0.0003 for DSS) or lymph node-positive HNSC patients (*n* = 238, HR = 1.932, *P* = 0.0009 for OS; *n* = 238, HR = 1.736, *P* = 0.0078 for PFI; *n* = 228, HR = 2.22, *P* = 0.0012 for DSS). The results demonstrated that IFI44 has important value in screening high-risk patients in local regional advanced HNSC (Table 1).

### Gene Ontology Analysis and Kyoto Encyclopedia of Genes and Genomes Pathway of IFI44 Co-Expression Genes in Head and Neck Squamous Cell Carcinoma

**FIGURE 3:**
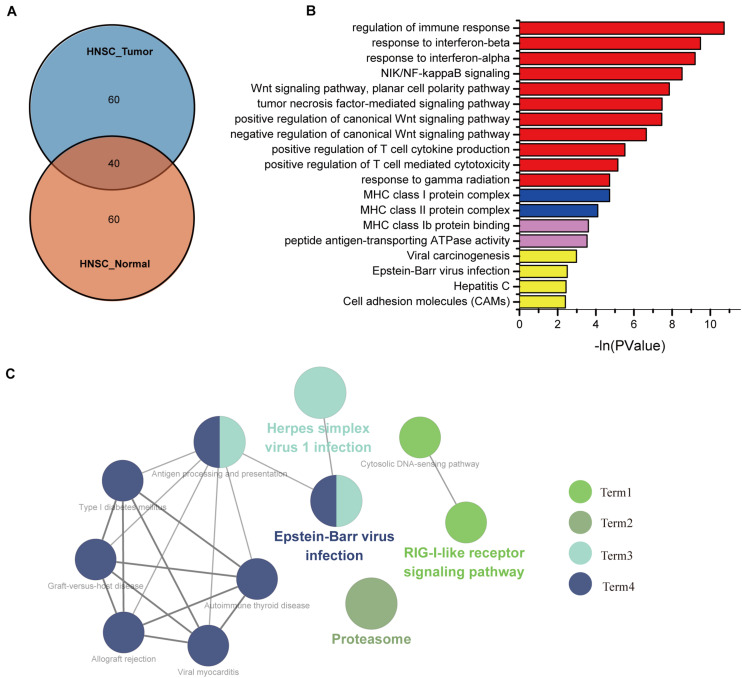
The biological process associated with IFI44 in head and neck squamous cell carcinoma (HNSC). **(A)** The Venn of IFI44 co-expression genes in HNSC tumor samples compared with normal samples. **(B)** Gene Ontology (GO) analysis including biological processes (BPs), cellular components (CCs), and molecular functions (MFs) was conducted using DAVID database. **(C)** Kyoto Encyclopedia of Genes and Genomes (KEGG) pathway analysis in Cytoscape.

To identify the underlying mechanisms of IFI44 involved in HNSC, we selected the top 100 genes from HNSC tumor samples and normal tissues through the GEPIA database, among which 60 genes were distinctly expressed in HNSC (Figure 3A and Supplementary Table 1). Using the gene set uniquely co-expressed with IFI44 in HNSC tumor samples, we conducted GO and KEGG pathways analyses (Figure 3B) *via* DAVID database. The present GO analysis was composed of biological processes (BPs), cellular components (CCs), and molecular functions (MFs). The biological processes of IFI44 co-expression gene set included type I interferon signaling pathway, interferon-gamma-mediated signaling pathway, response to interferon-gamma, response to interferon-beta, NIK/NF-κB signaling, Wnt signaling pathway, planar cell polarity pathway, tumor necrosis factor-mediated signaling pathway, positive regulation of canonical Wnt signaling pathway, negative regulation of canonical Wnt signaling pathway, positive regulation of type I interferon-mediated signaling pathway, and response to gamma radiation. Cellular components enriched in the IFI44 co-expression gene set of HNSC included Major Histocompatibility Complex (MHC) class I protein complex, nucleoplasm, nucleolus, and MHC class II protein complex. As to molecular function, the results consisted of protein binding, ATP binding, and MHC class Ib protein binding. The largest counting results from the KEGG pathway analysis were cell adhesion molecules (CAMs), Epstein–Barr virus (EBV) infection, viral carcinogenesis, hepatitis C, antigen processing and presentation, and Retinoic acid-inducible gene (RIG)-I-like receptor signaling pathway. The KEGG pathway analysis in Cytoscape also exhibited that IFI44 was associated with the process of EBV infection (21, 22), which was referred to the progression of nasopharyngeal carcinoma (NPC) (Figure 3C). Results above revealed that IFI44 was closely correlated with related mechanisms regarding tumor formation and progression.

### Gene Set Enrichment Analysis for Immune Signature in Head and Neck Squamous Cell Carcinoma

**FIGURE 4:**
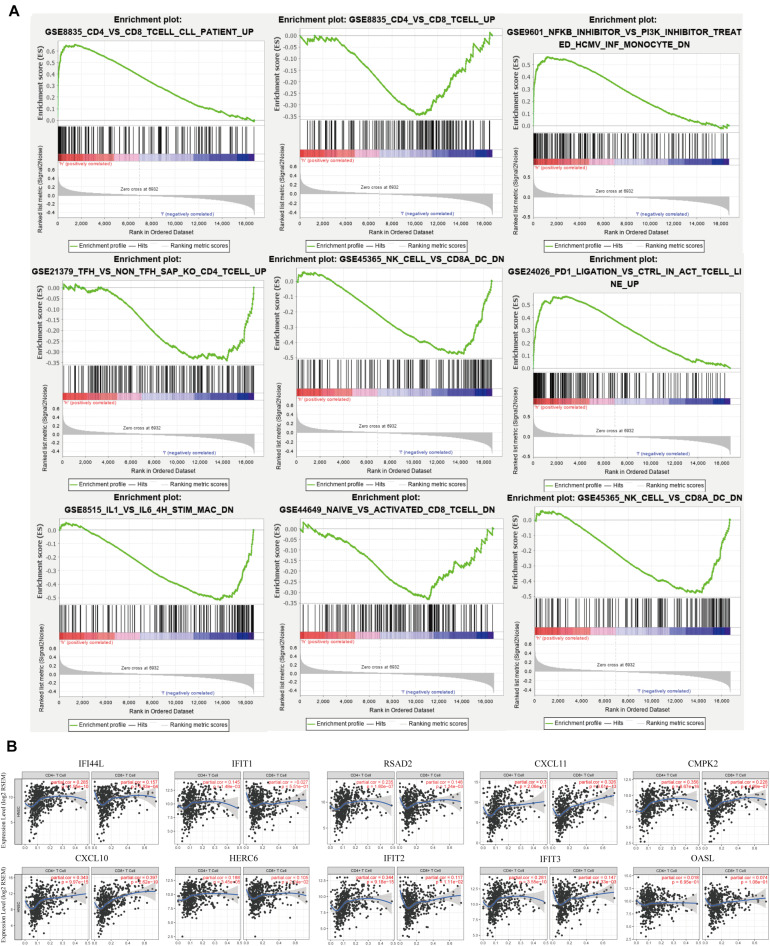
Gene set enrichment analysis (GSEA) of The Cancer Genome Atlas (TCGA) head and neck squamous cell carcinoma (HNSC) immune signature gene sets. **(A)** GSEA results enriched in IFI44-high expression group vs. IFI44-low expression group (http://software.broadinstitute.org/gsea/downloads.jsp). **(B)** The correlation between enriched immune genes (Top 10 enriched genes from GSE8835) with CD4^+^ cell vs. CD8 cell using Tumor Immune Estimation Resource (TIMER) database.

As signaling pathways of the immune response were acquired *via* the GO and KEGG analyses, we further performed GSEA to clarify the impact of IFI44 on immune function in HNSC. We divided 520 TCGA HNSC tumor samples into two groups according to IFI44 mRNA expression levels, and took C7 immune signature as a background gene set to conduct GSEA. After filtering with the standard of FDR < 0.25, Norm *P* < 0.05, part of the results were exhibited in Figure 4A. A total of 285 immune gene sets were enriched in IFI44-high expression group (Supplementary Table 2). Expression of IFI44 in GSE8835 was associated with the amount of CD4^+^ cells and CD8^+^ cells infiltrating in the tumor section, and 66 genes were distinguished as enriched immune signature genes in IFI44-high expression group in HNSC. To verify that IFI44 was closely related to the infiltration of CD4^+^ T lymphocytes in the tumor area, we then explored the relationship between 66 core genes and CD4^+^/CD8^+^ cells using TIMER database (Figure 4B). The CD4^+^ cell was known to be correlated with poor prognosis compared with CD8^+^ cells in patients with cancer (5, 23, 24). Figure 4B shows that the top 10 of genes upregulated in the IFI44-high expression group have a more positive correlation with CD4^+^ cells in HNSC. The results would explain the potential reason for poor survival in IFI44-high expression group.

### The Different Patterns of Immune Infiltration Between the IFI44-High Expression Group and IFI44-Low Expression Group in Head and Neck Squamous Cell Carcinoma

**FIGURE 5:**
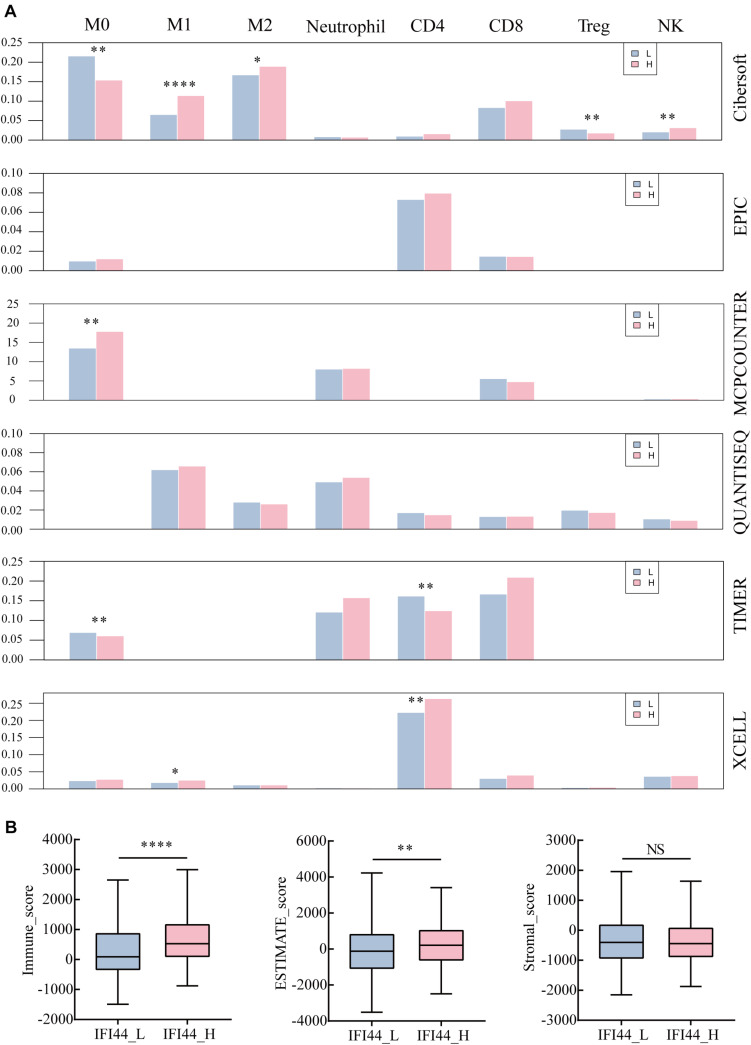
The different immune infiltration in IFI44-high expression group and IFI44-low expression group. **(A)** The immune infiltration of analysis across six databases between IFI44-high expression group (*n* = 261) and IFI44-low expression group (*n* = 261). M0 (macrophage M0), M1 (macrophage M1), M2 (macrophage M2), CD4 (CD4^+^ T cell), CD8 (CD8^+^ T cell), Tregs (regulatory T cells), NK (natural killer cell). **(B)** The immune infiltration score including immune score, stromal score, and estimate score in IFI44-high expression group (*n* = 261) compared with IFI44-low expression group (*n* = 261). The analysis was conducted in ESTIMATE database (*P < 0.05; **P < 0.01; ***P < 0.001; ****P < 0.0001).

To investigate the profile of immune infiltration in HNSC according to IFI44 expression, we collected multiple immune cell data from TIMER, CIBERSORT, quanTIseq, xCell, MCPCounter, and EPIC database. In terms of immune infiltration in tumor tissues, there was an obvious distinction between IFI44-high and IFI44-low expression groups (Figure 5A and Supplementary Table 3). IFI44-high expression group was infiltrated with more neutrophils, natural killer (NK) cells, macrophage M1, macrophage M2, Th1 and Th2 CD4^+^ T cells, CD8^+^ effector memory T cells, and plasmacytoid dendritic cells in the TME. And the proportions of macrophage M0, B cells, regulatory T cells (Tregs), endothelial cells, hematopoietic stem cells, and CD4^+^ naive T cells were significantly higher in IFI44-low expression group.

Further, we also evaluated the tumor purity and immune infiltration using the ESTIMATE algorithm between two groups (IFI44-high expression group and IFI44-low expression group) in HNSC (Figure 5). All were shown in Figure 5B that the IFI44-high expression group gained a higher immune score and ESTIMATE score than the IFI44-low expression group, which implied that the immune infiltration in HNSC has closely associated with IFI44 expression (Figure 5B). Although some discrepancies were observed across different databases, the results of calculations would sill explain the remarkable difference of immune infiltration between IFI44-high and -low expression groups (Figure 5A and Supplementary Table 3).

### The Genetic and Epigenetic Mechanisms Associated With IFI44 mRNA Expressions

**FIGURE 6:**
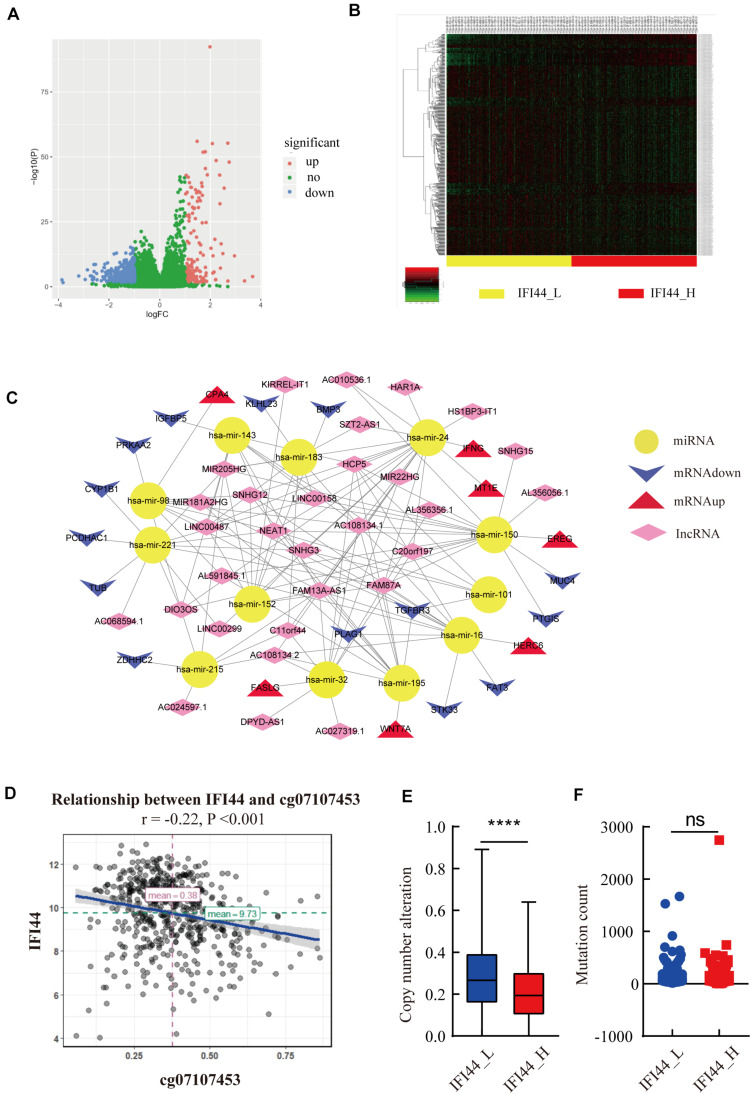
The upstream analysis associated with IFI44 overexpression in head and neck squamous cell carcinoma (HNSC). **(A–C)** The competing endogenous RNA (ceRNA) network associated with IFI44 expression. **(D)** The relationship between IFI44 expression and methylation level. **(E,F)** The copy mutation alteration and mutation count of IFI44-high expression group vs. IFI44-low expression group.

Since IFI44 mRNA expression had a profound effect on HNSC, we tried to illustrate the underlying mechanisms associated with gene transcription. DNA copy number variation and mutation counts are important factors affecting gene expression (25). The significant distinction between IFI44-high and IFI44-low expression groups was observed in copy number variation counts, but no significant difference was found in mutation counts (Figures 6E,F).

Moreover, DNA methylation was also an important mechanism regulating gene mRNA expression (26). Thus, we analyzed the relationship between IFI44 methylation and mRNA transcription sites. Results exhibited the obviously inverse correlation between IFI44 mRNA and methylation site of cg07107453 (Figure 6D; *r*_Pearson_ = −0.22, *P* < 0.001). Besides, ceRNA also had an essential influence on human carcinogenesis through affecting mRNA expression by competitively binding to common miRNA response elements (27, 28). CeRNA network mainly includes protein-encoding gene mRNA, LncRNA, pseudogenes, and circular RNA. In our study, we screened 426 LncRNAs (FC > 1, *P* < 0.05) that were markedly upregulated in IFI44-high expression group, 91 miRNA (FC > 1, *P* < 0.05), and 631 genes (log2|FC| > 1, *P* < 0.05) that were differentially expressed in the two groups. Then, we distinguished the interaction from upregulated miRNAs and LncRNAs based on miRcode database^10^. As a result, we gained 136 pairs in the ceRNA regulatory network, where all targeted genes were differentially expressed in IFI44-high expression group compared to IFI44-low expression group (Figures 6A,B and Supplementary Table 4). Interestingly, hsa-mir-150 has emerged in our network, a known overexpressed miRNA in NPC patients, which was closely related to 15 upregulated LncRNAs in the IFI44-high expression group. This network mainly showed the potential ceRNAs involved in the carcinogenic process of IFI44 in HNSC.

## Discussion

Interferon-induced protein 44 is a gene coding protein, engaging in the formation of microtubular structures. In the past years, researches have shown that IFI44 is one of the significant participants in immune response in autoimmune disease, HIV, and hepatitis diseases (9, 12, 29). However, studies about the prognostic value of IFI44 in cancers are still inadequate. As an important immune-related gene, IFI44’s potential value in tumor formation and progression is worthwhile to be disclosed.

In a previous study, the investigation provided preliminary evidence that high expression of IFI44 was associated with poor prognosis in HNSC and other types of cancer. First of all, IFI44 protein was principally located in the cytoplasm of HNSC cells. Higher expression of IFI44 was detected in the tumor section than normal tissues both at mRNA and protein levels (Figures 1A–E). Besides, HNSC patients with negative HPV infection that were related to poor prognosis expressed promoted IFI44 at the mRNA level compared with HPV-positive patients (Figure 1A). In new tumor occurrence after initial treatment of HNSC, IFI44 mRNA expression was found significantly higher than no recurrent cases (Figure 1B). Further, the survival time of the IFI44-high expression group was shown to be shorter than that of the IFI44-low expression group, especially in locally regional advanced patients whose clinical stage included lymph node-positive and Stage III/IV, suggesting the vital value of IFI44 acting as a prognosis biomarker in HNSC patients (Figure 2 and Table 1). In other types of cancer in TCGA, the abnormal expression of IFI44 was observed in BLCA, BRCA, CHOL, COAD, ESCA, KICH, KIRP, LIHC, LUAD, LUSC, and STAD tumor and paired normal samples. The level of IFI44 expression was also correlated with shorter DFS in LGG, PRAD, UVM, and UCS and shorter OS in LGG, UVM, and THYM (Supplementary Figure 1). In contrast, higher expression of IFI44 samples exhibited longer OS months in SKCM. Results above concluded that IFI44 acted as an oncogene in HNSC and functioned heterogeneously in tumor formation and progression.

Although ICB therapy is prevalent in the treatment of HNSC, the benefit is still limited due to the proper high charge and low objective response rate (30). Thus, it is urgent to search latent targets and formulate strategies selecting superior population to amplify the antitumor efficacy of ICB (31). Crucially, IFI44 was reported to participate in immune response, indicating the viability to mediate the immune response (12). To further reveal the possible biological functions of IFI44 in HNSC, KEGG and GO analyses of 60 IFI44 co-expression genes were performed (Supplementary Table 1). The results presented that the IFI44 co-expression gene set closely participated in multiple carcinogenesis and immune infiltration biological processes, particularly in type I interferon signaling pathway, immune response, NIK/NF-kappaB signaling and Wnt signaling pathway, and EBV infection. Also, the differentially expressed gene set *via* GSEA from GSE8835 dataset suggested that IFI44 was associated with an increased amount of CD4^+^ cells but lower infiltration of CD8^+^ cells in HNSC (24). By using TIMER database, the top 10 of GSE8836 core genes were identified as the closely positive relevance to CD4^+^ cells than CD8^+^ cells in HNSC. The results above showed that upregulation of IFI44 in HNSC would have a positive correlation with CD4^+^ cells, which referred to poor immune checkpoint therapy efficacy in multiple cancers.

Additionally, our study initially found that IFI44 participated in the adjustment of innate immune components. Tumor-associated macrophages mainly composed of M1 and M2 macrophage subtypes are important components of the tumor immune microenvironment in HNSC (32–34). However, subgroups of macrophages M1 and M2 have diverse functions within the TME, where M1 is constructive for the organism to resist inflammation and inhibit cancer development (35). According to our research, both a higher degree of macrophage M1 and macrophage M2 were observed in the tumor section of the IFI44-high expression group. Besides, the IFI44-high expression group showed more infiltration of neutrophils, which was associated with poor curing efficacy in cancer therapy (36). Finally, the immune infiltration and tumor purity level were evaluated through the ESTIMATE database, and the results further confirmed that increased IFI44 expression exhibited higher immune score and ESTIMATE score, which implied its intrinsic value in prediction and efficacy in immune checkpoint therapy. It is well recognized that the occurrence and development of tumors are not only related to tumor cells but also affected by the TME (37). All data demonstrated that IFI44 was related to immune infiltration in the TME.

Several studies have manifested that tumor formation is a complex process modulated by multiple factors at genetics and epigenetics level (38, 39). Our discovery of mechanisms regulating IFI44 expression mainly focused on methylation, copy number alteration (CNA), mutation count, and ceRNA network. Firstly, data analysis showed that the IFI44-high expression group contained more CNA count than IFI44-low expression group, while mutation count exhibited no valid difference between the two groups (Figure 6E). Secondly, the ceRNA network associated with IFI44 expression consisted of 12 miRNAs, 30 LncRNAs, and 21 mRNAs in HNSC, among which all LncRNAs were upregulated in the IFI44-high expression group. Hsa-mir-150 was downregulated in the IFI44-high expression group and was reported to participate in resistance to radiotherapy in NPC (40). This network mainly showed the potential ceRNAs involved in the carcinogenic process of IFI44 in HNSC.

## Conclusion

In summary, IFI44 was overexpressed in HNSC and correlated with poor outcomes in several types of tumors, especially in lymph node-positive and locally regional HNSC patients. Further, the expression of IFI44 was associated with the portions of neutrophils and M0, M1, and M2 macrophages in the tumor section. Mechanisms regarding the modulation of IFI44 expression were speculated to attribute to copy number variation count and DNA methylation, which need further research to verify. This study provides original knowledge that IFI44 plays a pivotal role in tumor immune infiltration and has a predictive value in the prognosis of HNSC patients.

## Data Availability Statement

Publicly available datasets were analyzed in this study. This data can be found here: https://xenabrowser.net/datapages/.

## Ethics Statement

The studies involving human participants were reviewed and approved by the Southern Hospital Ethics Committee. The patients/participants provided their written informed consent to participate in this study.

## Author Contributions

HP, XQW, and WQH designed this study. JG reviewed and modified this draft manuscript. All authors contributed to the article and approved the submitted version.

## Conflict of Interest

The authors declare that the research was conducted in the absence of any commercial or financial relationships that could be construed as a potential conflict of interest.

## Footnotes

1 https://www.ncbi.nlm.nih.gov/

2 https://portal.gdc.cancer.gov/

3 https://www.ncbi.nlm.nih.gov/geo/

4 https://genome.ucsc.edu/

5 https://www.cbioportal.org/

6 http://gepia.cancer-pku.cn/index.html

7 https://david.ncifcrf.gov/summary.jsp

8 https://cytoscape.org/

9 http://timer.cistrome.org/

10 http://mircode.org/

## Supplementary Material

Supplementary Figure 1 The different expressions of IFI44 in multiple cancers at the protein level.

Supplementary Figure 2 The prognostic potential of IFI44 in The Cancer Genome Atlas (TCGA) cancers. **(A–E)** Disease-free survival (DFS) of two different expression levels of IFI44 in head and neck squamous cell carcinoma (HNSC), brain lower grade glioma (LGG), prostate adenocarcinoma (PRAD), uveal melanoma (UVM), and uterine carcinosarcoma (UCS). **(F–I)** Overall survival (OS) of two different expression levels of IFI44 in LGG, thymoma (THYM), skin cutaneous melanoma (SKCM), and UVM.

## Abbreviations

IFI44: interferon-induced protein 44
HNSC: head and neck squamous cell carcinoma
CDS: coding sequence
BLCA: bladder urothelial carcinoma
BRCA: breast invasive carcinoma
CHOL: cholangiocarcinoma
COAD: colon adenocarcinoma
ESCA: esophageal carcinoma
KICH: kidney chromophobe
KIRP: kidney renal papillary cell carcinoma
LIHC: liver hepatocellular carcinoma
LUAD: lung adenocarcinoma
LUSC: lung squamous cell carcinoma
STAD: stomach adenocarcinoma
LGG: brain lower grade glioma
PRAD: prostate adenocarcinoma
UVM: uveal melanoma
THYM: thymoma
SKCM: skin cutaneous melanoma
OS: overall survival
DSS: disease-specific survival
PFI: progression-free interval
DFS: disease-free survival
NK cells: natural killer cells
CNA: copy number alteration
CeRNA: competing endogenous RNA
LncRNA: long-chain non-coding RNA
FDR: false discovery rate
HR: hazard ratio
CI: confidence interval
FC: fold change.
