# Supplementary figures and images for: Interferon-Induced Protein 44 Correlated With Immune Infiltration Serves as a Potential Prognostic Indicator in Head and Neck Squamous Cell Carcinoma
Source: Front Oncol. 2020 Oct 6;10:557157. doi: 10.3389/fonc.2020.557157 (PMC7573551; doi:10.3389/fonc.2020.557157)

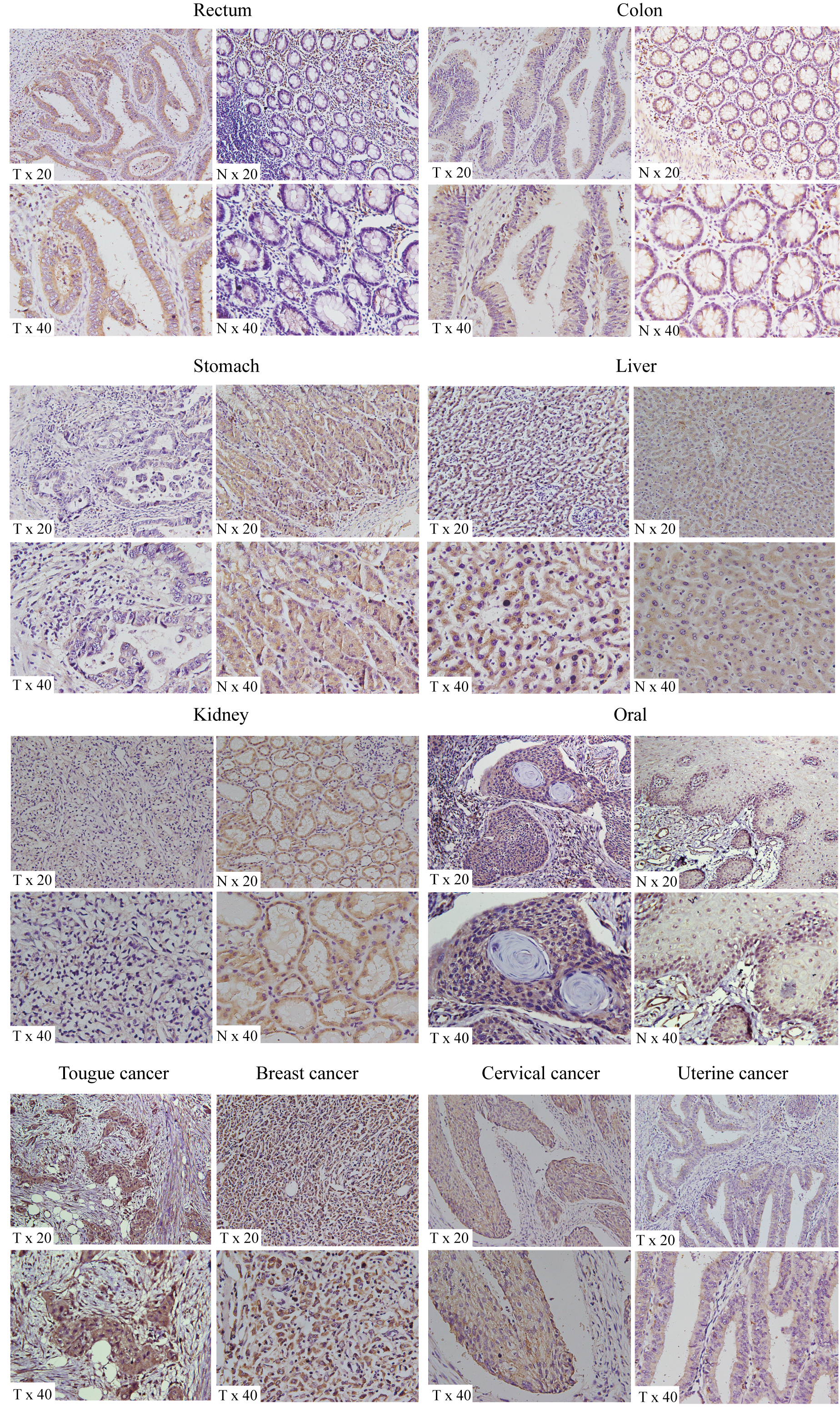

Supplement: Supplementary Figure 1 — The different expressions of IFI44 in multiple cancers at the protein level. [file Image_1.PNG]

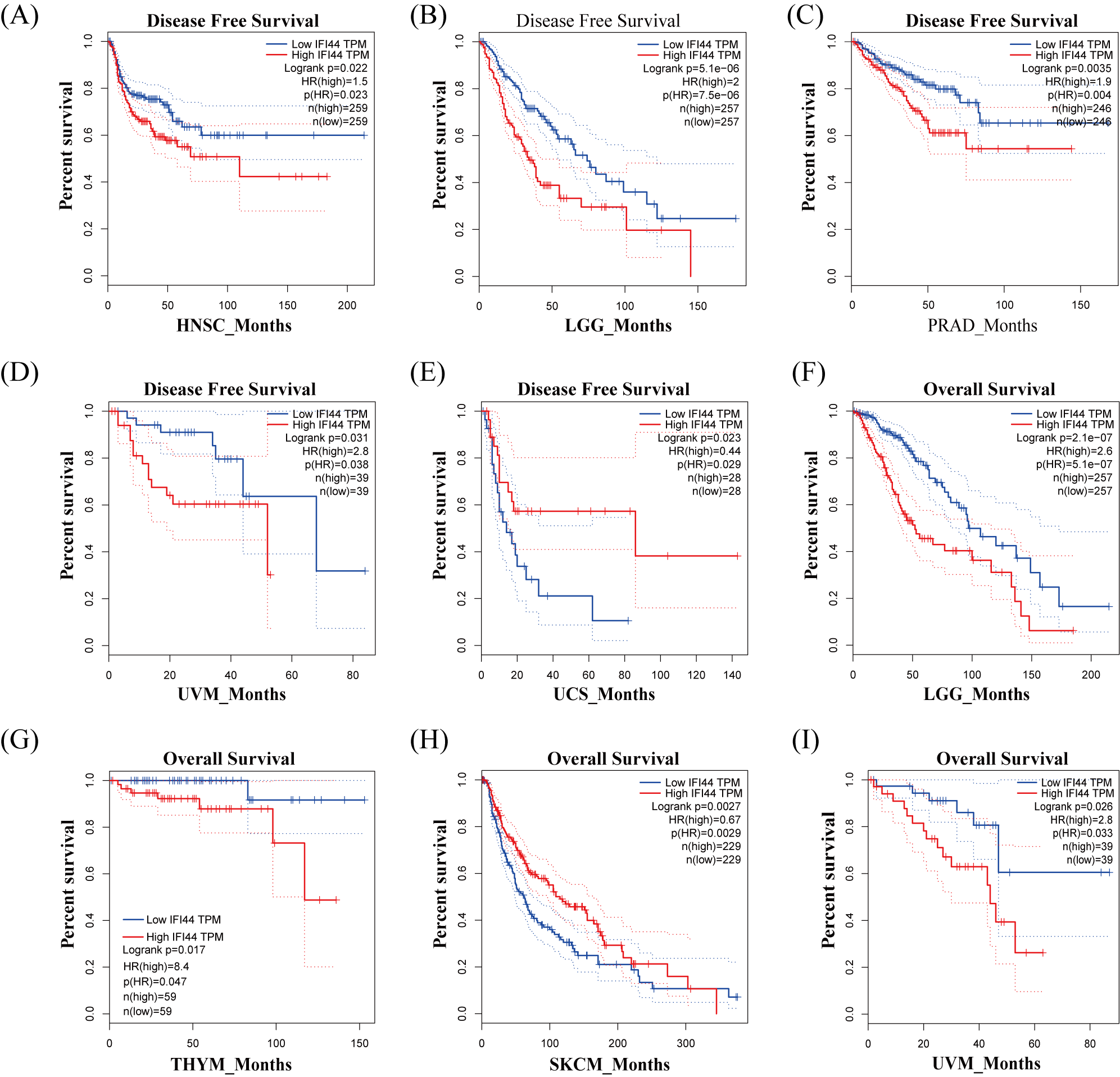

Supplement: Supplementary Figure 2 — The prognostic potential of IFI44 in The Cancer Genome Atlas (TCGA) cancers. (A–E) Disease-free survival (DFS) of two different expression levels of IFI44 in head and neck squamous cell carcinoma (HNSC), brain lower grade glioma (LGG), prostate adenocarcinoma (PRAD), uveal melanoma (UVM), and uterine carcinosarcoma (UCS). (F–I) Overall survival (OS) of two different expression levels of IFI44 in LGG, thymoma (THYM), skin cutaneous melanoma (SKCM), and UVM. [file Image_2.TIF]
